# Performance assessment of phylogenetic inference tools using PhyloSmew

**DOI:** 10.1093/bioadv/vbaf300

**Published:** 2025-11-23

**Authors:** Dimitri Höhler, Julia Haag, Alexey M Kozlov, Benoit Morel, Alexandros Stamatakis

**Affiliations:** Computational Molecular Evolution, Heidelberg Institute for Theoretical Studies, Baden-Württemberg 69118, Germany; Computational Molecular Evolution, Heidelberg Institute for Theoretical Studies, Baden-Württemberg 69118, Germany; Computational Molecular Evolution, Heidelberg Institute for Theoretical Studies, Baden-Württemberg 69118, Germany; Computational Molecular Evolution, Heidelberg Institute for Theoretical Studies, Baden-Württemberg 69118, Germany; Institute for Theoretical Informatics, Karlsruhe Institute of Technology, Baden-Württemberg 76131, Germany; Computational Molecular Evolution, Heidelberg Institute for Theoretical Studies, Baden-Württemberg 69118, Germany; Institute for Theoretical Informatics, Karlsruhe Institute of Technology, Baden-Württemberg 76131, Germany; Institute of Computer Science, Foundation for Research and Technology—Hellas (FORTH), Crete GR-70013, Greece

## Abstract

**Motivation:**

The performance of phylogenetic inference tools is commonly evaluated using simulated as well as empirical sequence data alignments. An open question is how representative these alignments are with respect to those, commonly analyzed by users. Using the RAxMLGrove database, it is now possible to simulate DNA and amino acid sequences based on more than 70 000 representative RAxML and RAxML-NG tree inferences on empirical datasets conducted on the RAxML web servers. This allows to assess the phylogenetic tree inference accuracy of various inference tools based on more realistic and representative simulated alignments.

**Results:**

To automate this process, we implement PhyloSmew, a tool for benchmarking phylogenetic inference tools. We use it to simulate ∼20 000 multiple sequence alignments (MSAs) based on representative empirical trees (in terms of signal strength) from RAxMLGrove. We subsequently analyze 5000 empirical MSAs from the TreeBASE database, to assess the inference accuracy of FastTree2, IQ-TREE2, and RAxML-NG. We find that on quantifiably difficult-to-analyze MSAs, all three tree inference tools perform poorly. Hence, the faster FastTree2 tool, constitutes a viable alternative to infer trees on difficult MSAs. We also find that there are substantial differences between accuracy results on simulated versus empirical data.

**Availability and implementation:**

The data underlying this article are available at https://github.com/angtft/PhyloSmew, https://cme.h-its.org/exelixis/material/accuracy-study/data.tar.gz.

## 1 Introduction

The field of phylogenetics aims to infer evolutionary relationships among species using molecular sequence data that are typically represented by phylogenetic trees. A plethora of methodological approaches exists, including distance-based methods [e.g. Neighbor Joining (NJ) (Saitou and Nei 1987)], Maximum Parsimony (MP) (Fitch 1971), and Maximum Likelihood (ML) (Felsenstein 1981), and Bayesian Inference (BI) (Rannala and Yang 1996, Yang and Rannala 1997, Mau and Newton 1997, Mau *et al.* 1999, Li *et al.* 2000). At present, ML and BI constitute the most widely used phylogenetic analysis methods as they rely on explicit stochastic models of sequence evolution. Note that, ML tree inference is computationally hard (Roch 2006) due to the superexponential growth in the number of possible tree topologies as a function of the number of taxa. Thus, current tools such as FastTree2 (Price *et al.* 2010), IQ-TREE2 (Nguyen *et al.* 2015, Minh *et al.* 2020), and RAxML-NG (Kozlov *et al.* 2019), rely on ad hoc search heuristics that strive to find “good” tree topologies with respect to the global optimum.

Accuracy assessments of trees inferred via these heuristics are typically based on collections of empirical and simulated datasets. To date, there exists no standardized approach or benchmark data collection for comparing tree inference tools. Instead, simulations are set up and empirical datasets are chosen ad hoc, based on criteria such as the number of sites, taxa, and proportion of missing data [see, for example, the respective papers describing FastTree2 (Price *et al.* 2010), IQ-TREE2 (Minh *et al.* 2020), and RAxML-NG (Kozlov *et al.* 2019)].

We examined the papers describing the aforementioned inference tools as well as a study comparing FastTree with RAxML (Liu *et al.* 2011) to characterize these ad hoc selection criteria. Then, we queried the RAxMLGrove database (Höhler *et al.* 2022) (RG) for datasets satisfying the criteria we characterize. We find that at most 27% of datasets present in RG do satisfy these criteria (Table 1). Therefore, we conclude that these criteria do not sufficiently represent the data typically being analyzed.

**Table 1.** Criteria for selecting empirical (emp) and simulated (sim) DNA datasets analyzed in different publications, including RAxML-NG (Kozlov *et al.* 2019), IQ-TREE (Nguyen *et al.* 2015), FastTree (Price *et al.* 2010), and the respective proportion of dataset diversity in RAxMLGrove that they cover (Cov.).

Source		Selection criteria	Results	Cov.
DNA RG		–	73.7k	100%
here: RG (sim)		<470 taxa, <18.7k patterns, model GTR+G	65.1k	88%
DNA TB		–	9.3k	100%
here: TB (emp)		<214 taxa, <3.4k patterns	8.5k	91%
Nguyen *et al.* (2015)		200–800 taxa, #sites/#taxa ≤4, #gaps ≤70%	8.3k	11%
Price *et al.* (2010) (emp)		500–23.9k taxa, 65–1.2k sites	1.1	2%
Price *et al.* (2010) (sim)		250-5k taxa	8k	11%
Kozlov *et al.* (2019) (emp)		36–1.8k taxa, 18.3k-37.3M sites	4.3k	6%
Liu *et al.* (2011) (sim)		≥1k taxa	1.4k	2%
Liu *et al.* (2011) (emp)	117–27.6k taxa	20.3	27%	

A recently published phylogenetic benchmarking tool and online service is PhyloBench (Spirin *et al.* 2024). It provides sets of empirical amino-acid MSAs, split into groups of 15, 30, or 45 sequences from the Pfam database (Mistry *et al.* 2021), on which the user must locally infer trees via the tool to be benchmarked. Then, the user uploads the trees via the web interface. PhyloBench subsequently computes topological distances [Robinson-Foulds distance (Robinson and Foulds 1981), Quartet distance (Smith 2022), Agreement distance (Dong and Kraemer 2004)] between the uploaded tree and trees inferred via six pre-defined inference tools.

In contrast to PhyloBench, our PhyloSmew benchmarking framework also allows for evaluating tools on simulated MSAs that are simulated along empirical trees from TreeBASE (Piel *et al.* 2009) or RAxMLGrove (Höhler *et al.* 2022) databases. As opposed to PhyloBench that only comprises amino acid MSAs, PhyloSmew also offers simulated and empirical DNA MSAs. Further, since we implemented PhyloSmew using Snakemake (Mölder *et al.* 2021), the resulting pipeline is parallelized and thereby allows to simultaneously benchmark any number of tree inference tools. PhyloSmew has two main use cases. First, precomputed trees can be used to evaluate a novel or modified tree inference tool. In this case, due to the Snakemake setup, the pipeline will solely reconstruct trees using the additional inference tool and compare them to the already available ones. Second, via a user configuration, PhyloSmew can select existing datasets from TreeBASE or simulate datasets using RAxMLGrove and run the complete analysis on a predefined set of tools to be benchmarked. Users can also import their own MSAs and omit the MSA selection step.

To demonstrate the utility of PhyloSmew, we conduct an example inference accuracy benchmark analysis for three widely used tree inference tools FastTree2 (Price *et al.* 2010), IQ-TREE2 (Minh *et al.* 2020), and RAxML-NG (Kozlov *et al.* 2019).

Liu *et al.* (2011) assessed the inference accuracy of RAxML (Stamatakis 2014) and FastTree (Price *et al.* 2009) using 1800 simulated and 10 empirical datasets. They find that RAxML outperforms FastTree on smaller datasets, with diminishing accuracy differences as dataset sizes increase. Similarly, Zhou *et al.* (2018) analyzed 19 empirical datasets (≤200 taxa, thousands of genes) and found that RAxML and IQ-TREE performed comparably, while FastTree performed worse. In contrast to these studies, here, we systematically classify datasets using Pythia (Haag *et al.* 2022, Haag and Stamatakis 2025), which quantifies dataset difficulty on a scale ranging from 0 (strong signal) to 1 (weak signal). Higher difficulty scores indicate larger uncertainty and induce a rugged tree space with multiple statistically plausible, yet topologically increasingly distinct, trees.

We analyze empirical and simulated DNA MSAs that cover at least 75% of the MSA properties contained in TreeBASE and RAxMLGrove (RG). We assess accuracy based on tree topologies, ML scores, and statistical plausibility. Our findings are consistent with preceding studies. However, they yield additional important insights into RAxML-NG, IQ-TREE2, and FastTree2 performance due to the larger data collection in conjunction with the Pythia-based difficulty assessment. This difficulty assessment allows us to identify difficulty ranges where these inference tools can be used interchangeably. Given that RG predominantly comprises DNA data (90% of datasets), we focus on DNA MSAs in our experimental setup and discussion. However, we also analyze a smaller set of amino acid (AA) datasets (see Supplement, available as supplementary data at *Bioinformatics Advances* online). PhyloSmew is available at https://github.com/angtft/PhyloSmew. Our pipeline can seamlessly be extended by additional phylogenetic inference tools and can thus contribute to conducting standardized phylogenetic inference performance studies. It can also be deployed for developing novel tree search heuristics.

## 2 Materials and methods

In this section, we outline our data selection and simulation strategies for our inference accuracy benchmark. We first describe how we deploy our RAxMLGrove database (RG) (Höhler *et al.* 2022) for MSA simulations and subsequently detail how we select datasets from the TreeBASE database (Piel *et al.* 2009).

### 2.1 RAxMLGrove database

We aim to generate realistic simulated MSAs that resemble empirical MSAs with respect to key statistical properties (e.g. number of sites, taxa, substitution rates, and gap proportions). To this end, we use the RAxMLGrove v.0.7 (RG) database, which contains over 70 000 anonymized phylogenetic trees and respective estimated model parameters inferred via the RAxML/RAxML-NG web servers [San Diego Supercomputer Center (Miller *et al.* 2010) and the Swiss Institute of Bioinformatics (https://raxml-ng.vital-it.ch)]. While the dataset origins (empirical versus simulated) and user backgrounds are unknown, the high volume of analyses (4000/month) suggests that RG is representative of general RAxML usage.

### 2.2 Simulated data

For simulating MSAs we use the recently published AliSim (Ly-Trong *et al.* 2022) simulator. AliSim can be parametrized with a phylogenetic tree, the desired simulated sequence length, a substitution model including its model parameters, nucleotide frequencies, and additional parameters to generate the MSA. We set those parameters according to an informed RG dataset selection strategy (see further below for details) via the corresponding functionality in the accompanying RAxMLGroveScripts (RGS) repository (https://github.com/angtft/RAxMLGroveScripts). RGS provides an SQLite database and appropriate Python scripts for seamless RG data access. In addition to dataset queries based on specific dataset characteristics (e.g. the number of taxa, or branch length variance of the tree), RGS provides a *generate* option to download datasets and simulate MSAs based on the model parameter estimates for that dataset using AliSim. Note that over 90% of the datasets are single partition MSAs. As RAxML-NG, FastTree2, as well as IQ-TREE2 are predominantly deployed for gene tree inference, we do deliberately not use more elaborate simulation tools that can emulate evolutionary processes beyond simple mutation and insertion/deletion events [for alternatives from population genetics, see for example Hoban *et al.* (2012)].

We generate simulated data based on the RG database version 0.7. As our goal is to assess ML tool inference behavior for *representative* datasets, we proceed as follows to select the datasets for our analysis:

1. We select trees inferred on DNA alignments under the General Time Reversible (GTR) model, as over 90% of trees in RG use this model
2. Datasets are chosen from the 95th percentiles of taxa and site patterns distributions, specifically with fewer than 470 taxa and 18.764 patterns, excluding excessively large outliers
3. Datasets are sorted by their site patterns-to-taxa ratio and divided into 20 000 buckets. One dataset is randomly selected from each bucket as a *representative*
4. For each representative, we simulate an MSA using the corresponding RGS functionality

This process selects 20 000 DNA datasets from RG that represent 88% of RG DNA data in terms of signal strength as approximated by the patterns-to-taxa ratio, and simulates MSAs based on the corresponding inferred ML trees and model parameters using AliSim. Following initial experiments, we excluded three datasets that could not be analyzed with RAxML-NG (v1.1.0) as they comprise partitions entirely containing undetermined characters, a feature that was removed in RAxML-NG v1.2.1.

Simulating MSAs with “realistic” gap patterns is challenging, as the “OVERALL_GAPS” entry in RG does not sufficiently capture the complexity of empirical MSAs. While the gap fraction alone exhibits a weak impact on dataset difficulty (Haag *et al.* 2022), the distribution of gaps does impact the number of distinct site patterns, which, in turn, correlates with difficulty (see Supplement, available as supplementary data at *Bioinformatics Advances* online). Initial experiments showed that omitting an insertion/deletion (indel) process in simulations yielded MSAs with over 20% fewer site patterns compared to RG entries. Thus, we followed Loewenthal *et al.* (2021) using the Zipfian distribution to model indels, optimizing its parameters via Bayesian methods [(Kushner 1964, Zhilinskas 1975, Mockus *et al.* 1978)] to minimize differences in sites, patterns, and gaps between simulated and empirical MSAs (details in Supplement, available as supplementary data at *Bioinformatics Advances* online).

### 2.3 Empirical data

We conducted experiments on 5000 MSAs from TreeBASE (Piel *et al.* 2009). The selection criteria that are based on the 95th percentiles of taxa and site patterns (analogous to the criteria used for simulating MSAs), yielded datasets with less than 214 taxa and 3475 site patterns, covering over 75% of TreeBASE’s dataset diversity (in terms of pattern/taxa ratio).

### 2.4 Pythia difficulty

In order to quantify the analysis difficulty of the underlying MSAs, we used the recently published Pythia (Haag *et al.* 2022, Haag and Stamatakis 2025) tool. Pythia uses a gradient-boosted trees model to predict a difficulty score between 0 and 1. The ground-truth label for a single dataset is based on $N_{\mathrm{all}}=100$ maximum-likelihood trees from independent tree searches using RAxML-NG. Let $RF_{\mathrm{all}}$ be the pair-wise RF-distance between all inferred trees, $N_{\mathrm{all}}^{*}$ the number of unique topologies, $N_{\mathrm{pl}}$ the number of plausible, that is, statistically indistinguishable trees (out of the *N* trees) using statistical tests implemented in IQ-TREE2, ${\mathrm{RF}}_{\mathrm{pl}}$ the pair-wise distance between all of these plausible trees, and $N_{\mathrm{pl}}^{*}$ the number of unique topologies among the plausible trees, the ground-truth difficulty is defined as:


$$\text{difficulty}=\frac{1}{5}\cdot [{\mathrm{RF}}_{\mathrm{all}}{+\mathrm{RF}}_{\mathrm{pl}}+\frac{N_{\mathrm{all}}^{*}}{N_{\mathrm{all}}}+\frac{N_{\mathrm{pl}}^{*}}{N_{\mathrm{pl}}}+(1-\frac{N_{\mathrm{pl}}}{N_{\mathrm{all}}})]$$

Thus, the difficulty score provides an intuition about the ruggedness of the tree space. A low score indicates a single, easy-to-find, likelihood peak while a high score reflects a rugged tree space comprising multiple, statistically equally plausible, yet topologically distinct trees. All prediction features are computed based on the underlying MSA and 100 parsimony starting trees generated by RAxML-NG. This yields the score fast and inexpensive to compute.

### 2.5 PhyloSmew benchmark

**Figure 1.**
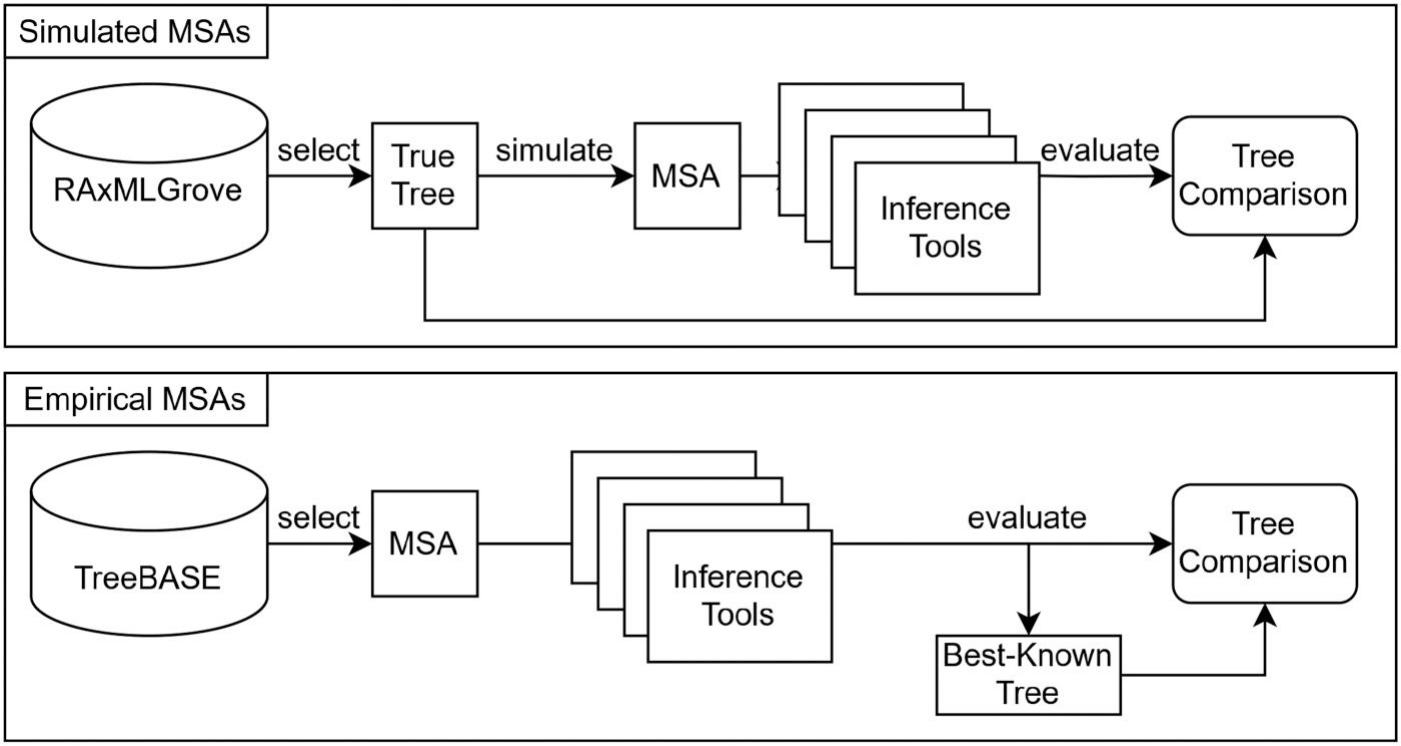
Flowchart of the PhyloSmew benchmark tool on simulated and empirical data.

We implemented PhyloSmew via a Snakemake (Mölder *et al.* 2021) pipeline. We selected, prepared, and generated the datasets as described above. The main steps of the pipeline are the following (see Fig. 1):

1. Query the database for datasets, sort by the ratio of site patterns to taxa, and assign to *n* buckets
2. Randomly select a dataset from each bucket
3. Run FastTree2, IQ-TREE2, and RAxML-NG inferences using the GTR model with Γ rate heterogeneity. Infer one RAxML-NG parsimony tree and evaluate all inferred trees with RAxML-NG to obtain comparable log likelihood (LnL) scores
4. Calculate pairwise Robinson-Foulds (RF) (Robinson and Foulds 1981), Normalized Tree (Zheng *et al.* 2015), and Quartet-distances (Estabrook *et al.* 1985), as well as LnL-differences between true trees or best-known ML trees (on empirical data) and the inferred ML trees. Run the Approximately Unbiased Test (Shimodaira 2002) [using CONSEL (Shimodaira and Hasegawa 2001)] on all trees (including the true tree)
5. Plot accuracy statistics

Thus, PhyloSmew comprises all main steps required to evaluate inference tools: It selects datasets, executes the tools, and computes result statistics, which can subsequently be visualized as simple plots such as the ones used in this manuscript, or, dynamically within a Dash app (https://github.com/plotly/dash). For tree inferences, we used the respective default parameters of each tool (see Supplement, available as supplementary data at *Bioinformatics Advances* online). Regarding the available substitution models, there only exist practical limitations when using PhyloSmew, since AliSim supports a wide range of models for all types of data. The practical limitations arise because (i) the vast majority of inferences stored in RG were conducted under the GTR+Γ(+I) model (+I models invariant sites), thus the corresponding MSAs are also simulated under this model, and (ii) the tree inference tools only support a subset of the available models. FastTree2, for instance, only supports the JC (Jukes-Cantor) and GTR substitution models for DNA data. One can circumvent these limitations by using custom MSAs and inference tools which support all desired models.

PhyloSmew compares inferred tree topologies to a given reference tree. For simulated MSAs, the reference tree is the “true” tree used for simulation. For empirical datasets, we use the best-known LnL tree as the “true” tree, following the approach of Zhou *et al.* (2018) and Price *et al.* (2010). That is, we consider the tree with the best-known LnL score as being the “true” tree. To determine this best-known tree, we executed a more thorough RAxML-NG search (100 independent tree searches, using 50 parsimony and 50 random starting trees). Thereby, we invest more computational effort into finding a best-known tree. If one of the other inference tools infers a tree with a higher LnL, we subsequently re-define the respective tree as the “true” tree.

The current benchmark implementation assumes that every inference tool will output one “best” tree. Thus, it is not intended to be used to compare trees inferred via Bayesian methods, although it is possible to draw a single tree [the MAP (maximum posterior probability) tree (Rannala and Yang 1996)] from the posterior tree sample. Thus, the benchmark is not technically limited to ML tools. Some of the metrics, however, such as the LnL differences or statistical AU tests (Shimodaira 2002), will compare trees based on their estimated likelihood. Therefore, ML tools will be favored as they directly strive to attain the highest possible likelihood.

Users can download the datasets from our study to assess the performance of other tools with respect to the precomputed trees. In this case, the pipeline will only execute tree searches with the newly integrated inference tool. To re-run PhyloSmew from scratch, users can use respective Snakemake cleanup command to remove intermediate files and specify data sources and selection criteria in the *config.yaml* file. PhyloSmew will then download the required empirical datasets (TreeBASE) or simulate them (RAxMLGrove) and execute the benchmark (Fig. 1). Users can generate analogous plots as presented here by using the scripts in *scripts.py.*

## 3 Results

Using PhyloSmew, we evaluated tree inference accuracy via the Robinson-Foulds (RF) distance (Robinson and Foulds 1981), the Normalized Tree Distance (NTD) (Zheng *et al.* 2015), and log-likelihood (LnL) differences. We quantified accuracy by comparing the true (simulated data) or best-known (empirical data) ML trees with the inferred trees. We also applied statistical significance tests via the Approximately Unbiased (AU) Test (Shimodaira 2002) and compared the number of times the inferred trees passed the tests with 95% confidence (compared to the best-known tree). We will refer to trees passing the test as *plausible* trees.

Our main focus is to analyze the performance of the three ML tools RAxML-NG, IQ-TREE2, and FastTree2. Due to the versatility of PhyloSmew, we also explore parsimony tree inferences and BIONJ inferences to attain a more comprehensive assessment.

After the analysis, we divided the results into 5 buckets based on MSA tree inference difficulty as predicted by Pythia (Haag *et al.* 2022). Every bucket (0 to 4) covers an interval of 0.2 difficulty units between 0.0 (easy dataset) and 1.0 (difficult/hopeless dataset).

Figure 3 shows the results of these experiments on empirical data (for numerical average values see Supplement, available as supplementary data at *Bioinformatics Advances* online).

**Figure 2.**
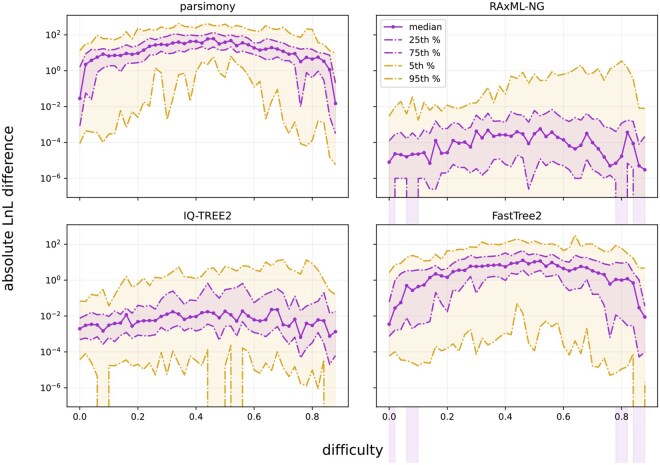
Absolute LnL score differences (note the log scale on the *y*-axis) between the trees found during tree inferences with RAxML-NG, IQ-TREE2, FastTree2, and parsimony (as estimated by RAxML-NG) and the best-known ML tree on TreeBASE data, as a function of the Pythia-based difficulty for the analyzed MSA. LnL scores were estimated via the RAxML-NG *evaluate* option.

On average, RAxML-NG yielded the highest LnL scores across all difficulty levels, followed by IQ-TREE2, while FastTree2 and parsimony performed worse. For easy (<0.2) and difficult (>0.8) MSAs, LnL differences from the best-known tree were typically within single-digit log-likelihood units (see Fig. 2). Median LnL differences remained within 4.8 units (0.16 excluding parsimony and BIONJ).

**Figure 3.**
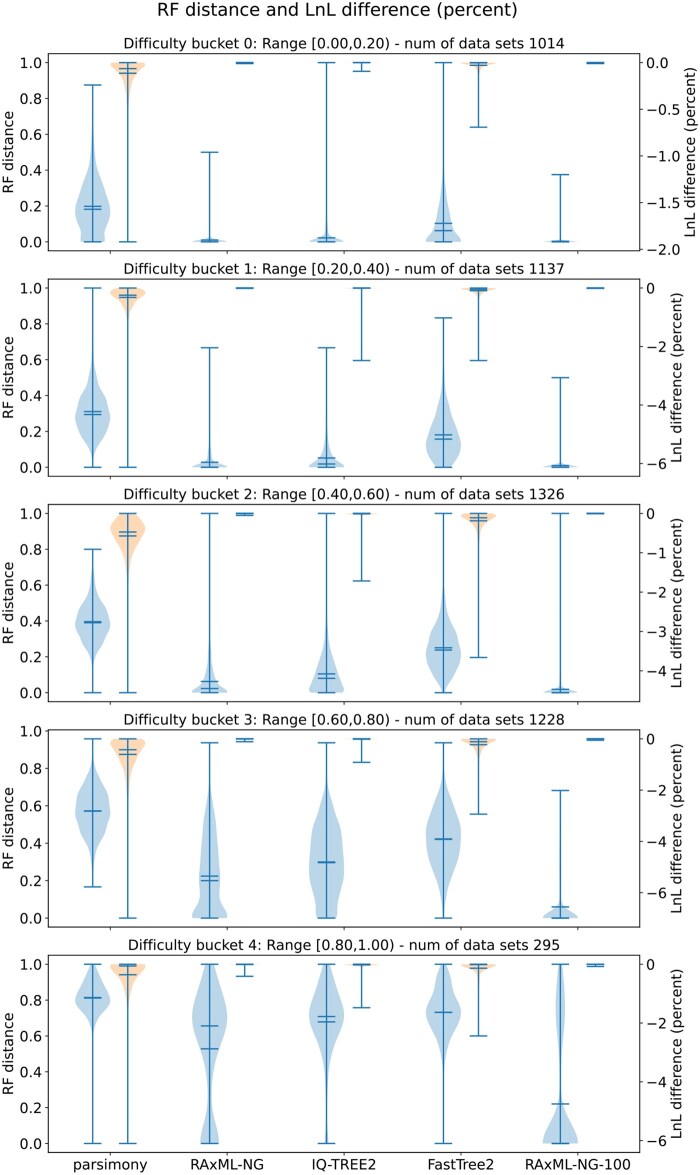
RF-distances (left), and relative log-likelihood (LnL) (estimated using RAxML-NG) score differences (right) of all inferred trees to the best-known ML tree on empirical TreeBASE datasets. “RAxML-NG-100” denotes the tree inferred by conducting 100 tree searches using RAxML-NG.

RF distances increased with difficulty (Spearman-ρ of 0.81, 0.74, 0.74, 0.60, 0.57, 0.23 for parsimony, FastTree2, IQ-TREE2, RAxML-NG, BIONJ, and RAxML-NG-100, respectively). RAxML-NG consistently found trees closest to the best-known tree, marginally followed by IQ-TREE2. This observation also applies to NTD values.

With respect to the AU test, RAxML-NG found the largest number of plausible trees across all buckets (>99% in buckets 0–3, 97.6% in bucket 4). IQ-TREE2 performed slightly worse (>98% in buckets 0–3). FastTree2 found plausible trees in 96% of bucket 0 cases, declining to 79% in bucket 2. Parsimony and BIONJ performed worse across all buckets (30%–79%).

For amino-acid data (see Supplement, available as supplementary data at *Bioinformatics Advances* online), RF- and NT-distance trends were analogous. However, in terms of LnL scores, we observed that FastTree2 rather unexpectedly outperformed IQ-TREE2 in four out of five buckets. In bucket 3, FastTree2 and parsimony yielded better average LnL scores than IQ-TREE2, due to outlier datasets, since IQ-TREE2 performed analogously to RAxML-NG (and substantially better than FastTree2 and parsimony) with respect to median LnL differences. Across all buckets, RAxML-NG inferred the highest proportion of plausible trees. IQ-TREE2 followed with the exception of bucket 3, where FastTree2 inferred a higher proportion of plausible trees (93% versus 81%).

**Figure 4.**
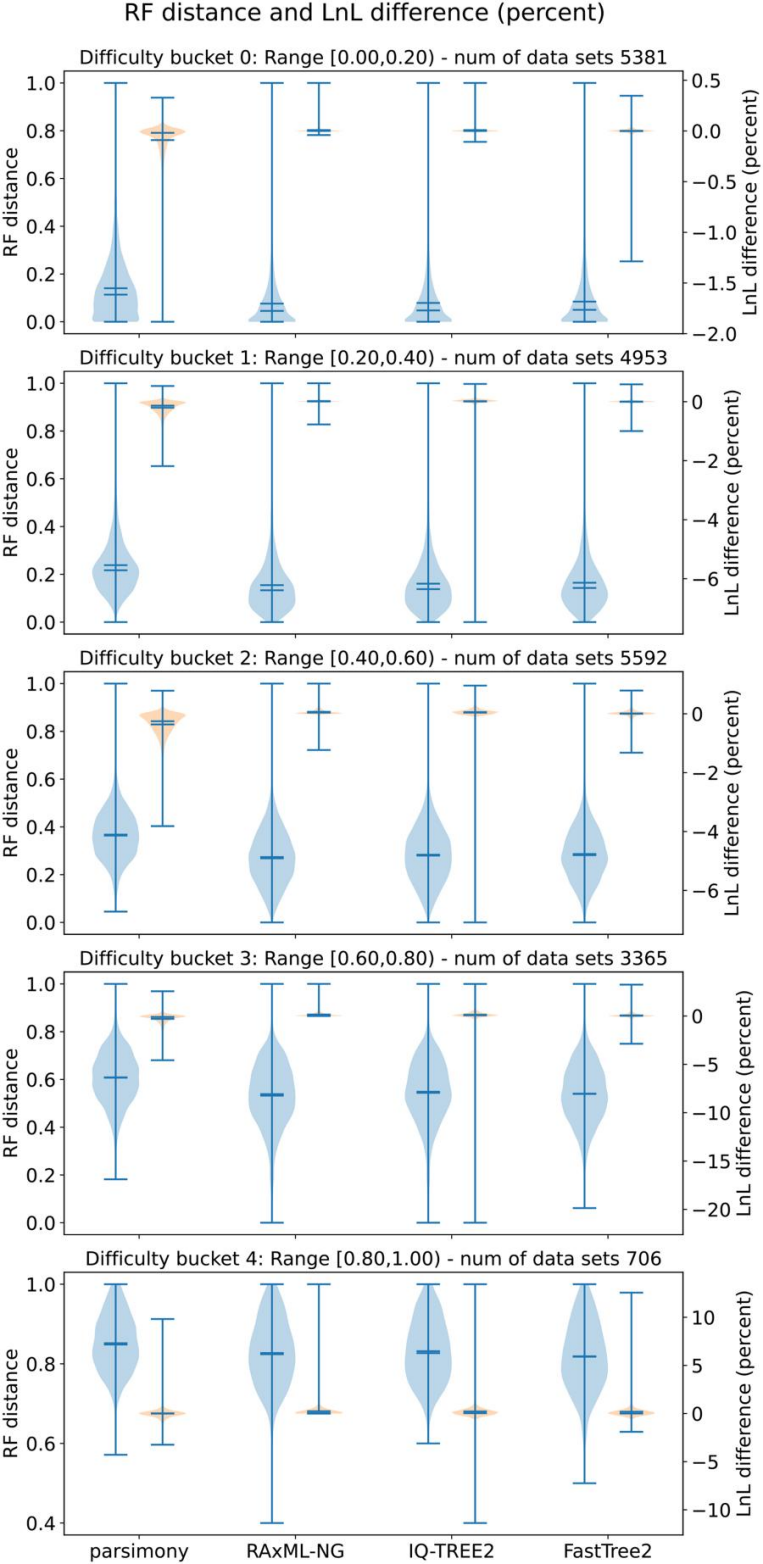
RF-distances (left), and relative log-likelihood (LnL) (estimated using RAxML-NG) score differences (right) of all inferred trees to the true tree on ∼20 000 simulated MSAs with RAxMLGroveScripts.

The results on simulated data as generated from RG trees are shown in Fig. 4 (for detailed average values see Supplement, available as supplementary data at *Bioinformatics Advances* online). On average, RAxML-NG finds the best LnL scores in all buckets. The differences to IQ-TREE2, however, are below 4 LnL units for all buckets. As expected, since the datasets are of finite length, these two tools find trees with a higher LnL score than the true tree in at least four out of five buckets on average (RAxML-NG: in all buckets). FastTree2 performed worse (by up to 6.3 LnL units), while parsimony and BIONJ were significantly worse (up to 79 and 2513 units, respectively).

RF distances exhibited insubstantial differences among RAxML-NG, IQ-TREE2, and FastTree2, with RAxML-NG performing best in most buckets while FastTree2 performed best in bucket 4. Parsimony and BIONJ performed worse. Again, RF distances increased with difficulty (Spearman-ρ ∼0.83 for all tools, except for BIONJ with 0.72).

NTDs showed a slightly distinct pattern (see Supplement, available as supplementary data at *Bioinformatics Advances* online). RAxML-NG and IQ-TREE2 performed similarly, but RAxML-NG outperformed FastTree2 in buckets 2–4 (NTD >0.004). This is more consistent with empirical data results. Parsimony outperformed BIONJ in all buckets.

AU tests showed that RAxML-NG and IQ-TREE2 performed analogously. On average, RAxML-NG, IQ-TREE2, and FastTree2 passed more statistical tests than the true tree (!). In bucket 4, the BIONJ tree was included in the set of plausible trees more frequently than the true tree.

Results on amino-acid MSAs were analogous (see Supplement, available as supplementary data at *Bioinformatics Advances* online). However, RAxML-NG and IQ-TREE2 consistently found trees with higher LnL scores than the true tree. This also holds for FastTree2 in buckets 0, 3, and 4, as well as parsimony in bucket 4.

RAxML-NG and IQ-TREE2 trees often yielded higher LnL scores than the true tree and passed more statistical tests. Yet, they do return inaccurate topologies (especially in buckets 3 and 4). This is not surprising, as the ML model is consistent when the number of sites goes to infinity. In practice, this might indicate that ML models overfit the data during the search (de Vienne *et al.* (2017) and favor incorrect topologies that maximize the LnL score. Being consistent with this observation, BIONJ finds more plausible trees than parsimony, despite yielding lower LnL scores.

For datasets with high difficulty (>0.8), inference tools encountered difficulties in recovering the true tree on simulated data (RF distances ≥0.7). On empirical data, RF distances to the best-known ML tree increased substantially (≥0.4) in difficult buckets. This suggests a weak signal strength that in turn induces a large number of statistically plausible, yet topologically distinct, trees. Due to the definition of the Pythia difficulty score, this behavior is expected, and shows that the difficulty measure is meaningful.

For low-difficulty datasets, reducing the number of independent tree searches in RAxML-NG may be reasonable, as increasing searches from 20 to 100 starting trees had negligible impact (<0.006 LnL units on average between RAxML-NG-100 and RAxML-NG). For difficult datasets, reducing the number of starting trees or deploying faster alternatives such as FastTree2 or parsimony [e.g. TNT (Giribet 2005)] may constitute a viable approach that does not deteriorate accuracy. Using Pythia predictions, which on average required 21.5% (median 6.8%) of the time needed for a single RAxML-NG tree inference (Haag *et al.* 2022), can therefore inform the setup of any computationally challenging phylogenetic analysis.

### 3.1 Differences between simulated and empirical data

We observed unexpected differences in RF distances between inferred trees and true trees on simulated and empirical datasets. On empirical datasets, differences in tree inferences were substantially more pronounced than on simulated datasets. This implies an interchangeability of tools, even outside the most difficult (>0.8) datasets. This may stem from approximations in inference and simulation models, as well as substantial differences in taxon numbers, site counts, site patterns, and gap proportions between TreeBASE and RG datasets (P≪.05, KS test).

Our method for simulating gap patterns might serve as a potential explanation. To investigate this, we conducted additional experiments using TreeBASE metadata, where empirical MSAs and their corresponding gap patterns are available. We initially simulated gapless MSAs based on the best-known ML trees and subsequently superimposed the original gap patterns before repeating our analyses.

Under this setting, RF distances more closely matched empirical data, with RAxML-NG yielding the most accurate trees while IQ-TREE2 and FastTree2 perform worse (see Supplement, available as supplementary data at *Bioinformatics Advances* online). The true tree had the highest LnL score on average, with RAxML-NG and IQ-TREE2 staying within 0.11 LnL units of it. FastTree2 remained below 4 LnL units. Despite these variations, trees inferred by RAxML-NG, IQ-TREE2, and FastTree2 were more often plausible than the true tree, with the exception of FastTree2 in the 0.2−0.6 difficulty range.

It remains unclear to which extent our gap simulation method contributes to the differences between TreeBASE and RG datasets. However, our findings are consistent with a prior study (Trost *et al.* 2024), showing that empirical gap patterns yield simulated MSAs that are more difficult to distinguish from empirical MSAs than simulated MSAs with simulated gap patterns. Differences in dataset difficulty distributions (see Supplement, available as supplementary data at *Bioinformatics Advances* online) between simulated and empirical data cannot fully explain these discrepancies. Other dataset properties that are currently not being captured by Pythia’s difficulty predictor, may also influence inference accuracy.

For our simulations, we used standard models of evolution as implemented in widely used sequence simulators, such as Dawg (Cartwright 2005), INDELible (Fletcher and Yang 2009), or AliSim (Ly-Trong *et al.* 2022). We specified the base frequencies, substitution rates, alpha parameters for the Γ model of site heterogeneity, gap rates, and evolutionary trees based on—what we assume to be—representative empirical datasets in RAxMLGrove. However, we did not account for issues such as, for instance, potential heterogeneous evolution across branches, or sequencing and alignment errors. At present, the impact of using more complex models on the degree of “realism” of resulting simulated MSAs and thus, on the resulting tree inferences, remains unclear.

Another potential bias stems from our “true” tree definition for empirical datasets as the best-known ML tree. This may favor RAxML-NG and IQ-TREE2. To test this, we applied the same approach to simulated data, selecting the highest-LnL tree from inferred trees and 100 additional RAxML-NG inferences (see Supplement, available as supplementary data at *Bioinformatics Advances* online). Under this setting, simulated results resemble empirical ones, with RAxML-NG and IQ-TREE2 yielding the most accurate trees and FastTree2, parsimony, and BIONJ performing worse.

Thus, defining the reference tree as being the best-known ML tree does introduce potential bias. However, differences between FastTree2, IQ-TREE2, and RAxML-NG were smaller under simulations than on empirical data. This suggests that additional, unaccounted-for factors, contribute to the observed discrepancies. Therefore, further research is required to evaluate tree inference tools. While ML methods aim to maximize LnL scores, their underlying goal is to reconstruct evolutionary relationships. Our results suggest that FastTree2, in many cases, offers a viable, faster alternative that avoids the potential over-optimization we observe for RAxML-NG and IQ-TREE2.

## 4 Conclusion

We presented a novel phylogenetic inference tool benchmarking pipeline called PhyloSmew. PhyloSmew offers a comprehensive evaluation pipeline for phylogenetic inference tools. It starts by selecting MSAs from TreeBASE or simulating MSAs via AliSim using RAxMLGrove to obtain representative simulation parameters. It then executes the predefined (but extensible) set of tree inference tools, and finally, evaluates and (optionally) visualizes the results. Due to the Snakemake (Mölder *et al.* 2021) implementation, the pipeline is parallelized and well-suited for deployment on computing clusters.

Using PhyloSmew, we assessed the tree inference accuracy of the three widely used phylogenetic inference tools RAxML-NG, IQ-TREE2, and FastTree2 on representative empirical as well as simulated DNA and amino acid MSAs from TreeBASE and RAxMLGrove, respectively. Compared to analogous studies such as by Liu *et al.* (2011) that claimed that FastTree2 performs almost as well as its much slower competitors on large datasets, that is, MSAs with thousands of taxa (which are uncommon in both, TB, and RG), we arrive at similar conclusions for specific groups of *commonly* analyzed datasets. Further, we are able to classify and differentiate among results by means of Pythia difficulty scores. Overall, we made the following observations:

First, we observed a discrepancy between our results on empirical and simulated MSAs, despite the fact that the simulated MSAs were generated using empirical parameter distributions and trees. Therefore, more research is required on generating more realistic synthetic datasets that behave analogously to empirical datasets with respect to the tree inference process.

Second, we observed a bias in topological distances when comparing inferred trees to the best-known tree (with respect to its LnL) instead of the true tree. The best-known tree might be considered as constituting the best hypothesis when the true tree is unknown [e.g. Zhou *et al.* (2018)]. Our experiments on simulated data suggest that topological tests tend to favor more thorough LnL optimization algorithms. However, we also note that some techniques for simulating gaps in an MSA can yield more “realistic-looking” MSAs (Trost *et al.* 2024), and that our specific method of gap generation might introduce additional biases. These factors should be considered when designing and interpreting accuracy tests.

Third, we observed that with increasing difficulty, as predicted by Pythia, the accuracy of all analyzed tools deteriorates and the accuracy differences between these tools diminish. This confirms that Pythia implements a meaningful measure for quantifying dataset difficulty in practice. It is important to note that an “easy” dataset according to Pythia does not necessarily imply a consistent reconstruction of the true tree. However, it appears to be more likely to recover the true tree according to our experiments. Thus, given the fact that the difficulty score is fast to compute (Haag *et al.* 2022)—compared to a typical analysis using RAxML-NG—we recommend applying Pythia before conducting phylogenetic analyses.

Finally, we find that on datasets exhibiting a high difficulty level (Pythia difficulty above 0.8), all analyzed tools can essentially be used interchangeably, according to our simulations. However, even on empirical data, where some tools clearly outperform others, we find that all analyzed tools are generally inaccurate. This means that resource-intensive computations could—and we would argue *should—*be avoided on such difficult or “hopeless” datasets. More specifically, one should critically assess the necessity of compute-intensive ML optimization routines, as they are performed by RAxML-NG and IQ-TREE2, especially when considering the fact that—due to potential biases in evaluations based on empirical data—it is not entirely clear, to which degree these tools are more accurate compared to faster competitors. Therefore, we conclude that PhyloSmew can indeed be used to conduct, analyze, and reproduce phylogenetic inference tool benchmark studies in a seamless and straight-forward manner. In our lab, we are actively using PhyloSmew for developing and evaluating novel RAxML-NG tree search heuristics. Additionally, we propose the development of adaptive and flexible heuristic search algorithms that can dynamically take into account the degree of difficulty and the properties of the dataset being analyzed. While preparing the current manuscript, our research group already developed a prototype of such an adaptive search strategy in RAxML-NG. Adaptive RAxML-NG takes the MSA difficulty into account, and specifically reduces the amount of starting trees for highly difficult datasets (among other optimizations) (Togkousidis *et al.* 2023). This leads to average speedups of at least a factor of 5 without substantially deteriorating the already low inference accuracy. For “What falleth, that shall one also push! […] And him whom ye do not teach to fly, teach I pray you-to fall faster!” (Nietzsche 1909).

As benchmarking phylogenetic inference tools constitutes a compute- and resource-intensive endeavor, we consider developing approaches to reduce the computational requirements of benchmarking runs. For instance, one may consider the following sub-sampling challenge: Out of the *x* datasets in our benchmark, can we select *y* representative ones such that y≪x and the results on these *y* datasets allow for an accurate accuracy and run-time prediction for the remaining ones? This will be particularly useful for developing ad hoc tree search heuristics that need to iteratively assess the impact of algorithmic changes.

## Supplementary Material

vbaf300_Supplementary_Data
