# Supplementary material for: Performance assessment of phylogenetic inference tools using PhyloSmew
Source: Bioinform Adv. 2025 Nov 23;5(1):vbaf300. doi: 10.1093/bioadv/vbaf300 (PMC12701799; doi:10.1093/bioadv/vbaf300)
Supplement: vbaf300_Supplementary_Data [file vbaf300_supplementary_data.pdf]

# Supplement: Performance Assessment of Phylogenetic Inference Tools using PhyloSmew

Dimitri Höhler[1], Julia Haag[1], Alexey M. Kozlov[1], Benoit Morel[1,2], and Alexandros Stamatakis[3,1,2]

[1]Computational Molecular Evolution group, Heidelberg Institute for Theoretical Studies, Heidelberg, Germany
[2]Institute for Theoretical Informatics, Karlsruhe Institute of Technology, Karlsruhe, Germany
[3]Institute of Computer Science, Foundation for Research and Technology Hellas, Crete, Greece

November 20, 2025

## 1 Dataset Simulations

We used AliSim [18] to simulate MSAs for our experiments. We executed AliSim by specifying the tree, the used model and estimated model parameters from selected RAxMLGrove datasets. For simulating gaps, AliSim offers the `--indel` and `--indel-size` parameters. The `--indel` parameter is a user-specified rate for generating insertions and deletions (so-called *indels*) at sites during the sequence simulation process. The indel lengths are drawn from distributions, which can be specified using the `--indel-size` parameter. Currently, AliSim supports the Geometric, Negative Binomial (NB), Zipfian, and Lavalette distributions originally proposed in [4], which can be separately set and selected for the insertion and deletion process. Different distributions also need to be parameterized differently (e.g., mean and variance for indel sizes for the Negative Binomial distribution). Thus, even if we use the same distribution for insertions and deletions, we need to set a total of 6 parameters, that is, the insertion and deletion rates as well as the respective means and variances of the insertion and deletion sizes to parameterize the NB distribution. An additional complication is that the sequence length parameter used by some simulators (e.g., AliSim) only defines the sequence length of the single starting sequence, on which the simulation is then carried out along the tree. Thus, when gaps are introduced during the simulation process, the overall MSA length will typically exceed the seed/root sequence length.

Under the assumption that a simulation under the 'correct' settings for these 6 parameters will result in a simulated MSA where the differences between the number of sites, number of patterns, and gap percentage of RG entries and simulated MSAs are minimal, we can define an evaluation function $d$ to quantify how well the simulated MSA matches the original MSA. Let $s_{orig}, p_{orig}, g_{orig}$ and $s_{sim}, p_{sim}, g_{sim}$ be the number of sites, number of patterns, and fraction of gaps in the RG entries and the simulated MSAs, respectively. Further, let $w_1, w_2, w_3$ be arbitrary, yet constant weights. We can then define $d$ as follows:

$$\begin{aligned} d = w_1 &\frac{abs(s_{orig} - s_{sim})}{s_{orig}} \\ + w_2 &\frac{abs(p_{orig} - p_{sim})}{s_{orig}} \\ + w_3 &abs(g_{orig} - g_{sim}) \end{aligned} \tag{1.1}$$

Since it is unclear how to set these parameters such as to minimize $d$ for a given simulated dataset, we resorted to Bayesian optimization [14, 33, 20], using the Python library skopt [8], and implemented the Bayesian Optimized iNdel seeKer (BONK) in RGS. Given $w_1, w_2, w_3$, a scaling factor $c_{seq}$, insertion and deletion size distributions, and a maximum number of optimization rounds $n_{opt}$, BONK uses the optimizer to iteratively explore the indel rates and size distributions to minimize $d$ for the simulated MSA. For the sake of simplicity, we arbitrarily chose (preliminary tests indicated no apparent reason to choose one distribution over another) the NB distribution for both insertion, and deletion sizes. We use $c_{seq}$ to define the sequence length range for the root sequence. As stated above, the length of the root sequence has to be chosen as a function of the gaps being inserted to minimize the difference between $s_{orig}$ and $s_{sim}$. The length interval is defined as $[s_{orig} \times (1 - c_{sim}), s_{orig} \times (1 + c_{sim})]$. Preliminary experiments have also shown that, apart from $n_{opt}$, the minimization of $d$ is strongly affected by the values of $w_1, w_2, w_3$. These weights penalize some property differences more than others, and since the number of sites, number of patterns, and gaps are correlated, it appears that choosing these weights is not trivial.

Thus, we deployed a second optimizer (results not shown) to optimize the weights with respect to the average $d$ of simulations based on random subsets of datasets from RG. We found that $w_1 = 9, w_2 = 10, w_3 = 1, c_{seq} = 0.7$ worked sufficiently well, and used these weights for all our simulations with BONK.

After implementing BONK, we discovered SpartaABC [16, 17], a tool which uses the Approximate Bayesian Computation method [25, 28] to estimate the indel rates and sizes of a given MSA. The authors use a far more sophisticated distance function than $d$, overall comprising 27 features. Since the original MSAs are not available in RG, we cannot compute their proposed distance function and include SpartaABC into RGS. Thus, our simulations were conducted with BONK. However, we followed the suggestions regarding SpartaABC (and references to empirical studies) and made adjustments to BONK: We set the explored

| | Mean | Median |
|---|---|---|
| sequence length (no gaps) | 0.00 | 0.00 |
| patterns (no gaps) | 0.19 | 0.15 |
| gaps (no gaps) | 0.13 | 0.08 |
| sequence length (after opt) | 0.01 | 0.00 |
| patterns (after opt) | 0.06 | 0.02 |
| gaps (after opt) | 0.14 | 0.10 |

Table 1: Normalized (absolute) MSA property differences between RG entries and simulated MSAs. We compare the trivial (no gap insertion) with the optimized simulation using BONK.

indel intervals to [0.0, 0.05], switched to the Zipfian distribution for indel sizes, and set the explored interval of the corresponding $a$ shape parameter to [1.001, 2].

In addition to the aforementioned procedures, we also use the presence/absence matrices for partitioned datasets, when available in RG. A presence/absence matrix is a 2-dimensional binary matrix denoting the presence of information (sequence data) for taxon $k$ at partition $l$. If there is no sequence data for taxon $k$ at partition $l$ we set $M[k, l] := 0$, and $M[k, l] := 1$ otherwise. After the simulation process, we therefore remove per-partition sequences from the MSA according to $M$. This is conducted before calculating $d$, such that the optimizer is aware of the gaps introduced by $M$.

**Simulation Quality**

We evaluated our BONK method for gap-aware simulations by comparing the absolute differences between the properties (i.e., number of sites, number of site patterns, and gap proportion) of the simulated MSA and the respective RG database entry. We show the normalized differences in Table 1. In the worst case, that is, when the optimizer is not able to find parameters improving upon trivial simulations, we obtain an MSA without gaps. On average, we are able to simulate MSAs with properties which are closer to those of the RG entries than under trivial simulations without an indel model in terms of pattern numbers. However, the difference in the gap proportions remained roughly the same - with the change that we now often insert more gaps than necessary.

We further compared the difficulty of the simulated and original MSAs using Pythia [6]. For this, we randomly selected a subset of 989 TreeBASE DNA datasets (note that the original empirical MSAs are required for difficulty prediction, which are available in TreeBASE but not in RG), using the same size-based dataset selection criteria as in the preceding experiments, inferred a RAxML-NG tree using 50 parsimony and 50 random starting trees under the GTR+$\Gamma$ model and used the inferred best-known ML tree, and model, to conduct the following distinct MSA simulations: (1) AliSim simulation without any indels; (2) AliSim simulation with the so-called mimicking function, which uses a speci-

| | Mean | Median | Stdev |
|---|---|---|---|
| (1) no gaps | 0.0799 | 0.0510 | 0.0892 |
| (2) AliSim mimick | 0.1052 | 0.0657 | 0.1138 |
| (3) BONK | 0.0868 | 0.0518 | 0.0955 |
| (4) (shortened) SpartaABC | 0.0881 | 0.0535 | 0.0980 |

Table 2: Absolute differences in difficulty between the original (empirical) MSAs and the simulated MSAs (based on 1000 datasets from TreeBASE, selected from 95-percentiles of taxa and site pattern numbers).

fiable (original) MSA to first infer a tree, then simulate an MSA, and subsequently superimpose the gap pattern from the original MSA to the simulated one; (3) BONK; (4) the SpartaABC tool, albeit with modifications to its internal pipeline. In SpartaABC, we removed the realignment step and reduced numbers of default burn-in and optimization rounds ($1,000/10,000$ instead of $10,000/100,000$ respectively) to reduce the overall computational time, as some of the MSAs required more than 24 hours for a single indel rate inference. After the analysis, we used the suggested indel parameters by SpartaABC to simulate 10 MSAs with AliSim and selected the MSA with the lowest distance (as defined in SpartaABC) to the original MSA. We are fully aware that we do not fully utilize the capabilities of the SpartaABC algorithm, but we include it here to provide an intuition of how this method could perform in terms of difficulty matching, since it might be a valuable addition to the RGS simulation procedure.

In Table 2, we observe the following: Overall, there are small absolute differences between (1), (3), and (4). One might argue that the differences of these three approaches fall within the margin of error of the difficulty prediction. Surprisingly, inserting no gaps at all yields the difficulty prediction that is closest to the original difficulty, albeit the number of patterns more closely matches the original when using (3) instead of (1), and the gap proportion errors of (1) and (3) with respect to the original are similar. (1) and (3) do not require additional information about the underlying MSA, other than the properties already available in RG. Method (3) requires $n_{opt} = 100$ steps, which means that it is at least 100 times slower than the single MSA simulation conducted by method (1). Method (4) requires an a priori computation of the gap features for the SpartaABC distance function. This could potentially be included into a future RG release, as it requires little additional memory to store these data and does not expose the original MSA to the public (i.e., it can directly be computed on the respective web-servers before being stored in RG). Method (2) is probably the most intuitive approach. Here, the distance $d$ is negligible on average (data not shown). This method performs worst in terms of difficulty differences. Method (2) also requires the complete original MSA (if using the default AliSim mimick function) or at least the gap matrix of the original MSA to be available (if using the already inferred best tree and model present in RG).

## 2 Tree Inference Commands

We used the following commands to execute the ML tools:
RAxML-NG

```
raxml-ng --msa assembled_sequences.fasta
    --model GTR+G --prefix [prefix]
    --seed [seed] --threads 4
    --force perf_threads
```

For partitioned datasets we substituted "–model GTR+G" with "–model [partition_file]" as all original partition files were available for empirical datasets. For simulated datasets, RG contains per-partition substitution model and MSA length parameters, based on which RGS simulates the MSAs and generates the partition files.

IQ-TREE2

```
iqtree2 -s assembled_sequences.fasta
    -m GTR+G -nt 4
    --prefix [prefix]
```

For partitioned datasets, we appended "-p [partition_file]".

FastTree2

```
FastTreeDbl -gtr -gamma
    -nt assembled_sequences.fasta
```

To the best of our knowledge, FastTree2 does not support partitioned analyses and therefore all analyses were conducted on unpartitioned MSAs. We could not evaluate some of the FastTree2 trees via RAxML-NG as they contained multifurcations. In that case, we resolved the multifurcations at random prior to LnL evaluation.

## 3 Statistical Data

In this Section, we present numerical statistical results of our experiments using the PhyloSmew pipeline on 5 inference methods: RAxML-NG [13], IQ-TREE2 [19], FastTree2 [23], parsimony [3] (estimated using RAxML-NG), BIONJ [5] (computed using IQ-TREE2). As described in the main manuscript, we split the results into 5 buckets according to the Pythia [6] difficulty estimation, with each bucket covering an interval of 0.2 in the range from 0 to 1.

### 3.1 DNA MSAs

Tables 3 to 6 show the average RF-distances, average Normalized Tree Distances (NTDs), average LnL differences to best-known ML tree, and the average num-

| Bucket | RAxML-NG 100 | RAxML-NG | IQ-TREE2 | FastTree2 | pars | BIONJ |
|---|---|---|---|---|---|---|
| 0 | **0.003077** | 0.010144 | 0.022360 | 0.103195 | 0.198072 | 0.258025 |
| 1 | **0.009620** | 0.027854 | 0.051710 | 0.180850 | 0.310706 | 0.402073 |
| 2 | **0.018650** | 0.062505 | 0.104724 | 0.250458 | 0.396037 | 0.454734 |
| 3 | **0.059761** | 0.224224 | 0.299874 | 0.420655 | 0.572774 | 0.550543 |
| 4 | **0.220675** | 0.528081 | 0.678354 | 0.731477 | 0.811495 | 0.789749 |

Table 3: Average RF distances to the best-known ML tree. Experiments conducted on DNA MSAs from TreeBASE. Tree inferences conducted under the GTR+G model.

| Bucket | RAxML-NG 100 | RAxML-NG | IQ-TREE2 | FastTree2 | pars | BIONJ |
|---|---|---|---|---|---|---|
| 0 | **0.000618** | 0.001288 | 0.005133 | 0.025151 | 0.039901 | 0.078214 |
| 1 | **0.000184** | 0.000905 | 0.008681 | 0.041476 | 0.066731 | 0.134297 |
| 2 | **0.000656** | 0.003144 | 0.013953 | 0.059018 | 0.088926 | 0.148481 |
| 3 | **0.001199** | 0.007009 | 0.019936 | 0.066324 | 0.093683 | 0.120723 |
| 4 | **0.008317** | 0.017023 | 0.030954 | 0.059614 | 0.071444 | 0.094786 |

Table 4: Average NTDs to the best-known ML tree. Experiments conducted on DNA MSAs from TreeBASE. Tree inferences conducted under the GTR+G model.

bers of passed statistical tests for experiments conducted on DNA MSAs from TreeBASE [22, 30]. The median values are shown in Tables 7 to 9.

Tables 10 to 13 show the same statistics for experiments conducted on simulated DNA MSAs using RAxMLGroveScripts with BONK gap insertions (19, 997 datasets).

Figure 1 and Tables 14 to 17 show the statistics for a subset of 1000 datasets of the simulated DNA MSAs. Here, we compared the tree inferences to the best-known-LnL tree, rather than to the true tree, to test for a potential bias.

| Bucket | RAxML-NG 100 | RAxML-NG | IQ-TREE2 | FastTree2 | pars | BIONJ |
|---|---|---|---|---|---|---|
| 0 | **-0.001236** | -0.006590 | -0.060168 | -3.007678 | -13.869848 | -309.091563 |
| 1 | **-0.008760** | -0.023973 | -0.599720 | -18.689795 | -66.144411 | -1085.846431 |
| 2 | **-0.008610** | -0.109468 | -1.893994 | -29.690353 | -82.940122 | -791.810745 |
| 3 | **-0.015275** | -0.211689 | -1.872020 | -20.250131 | -43.755156 | -314.300644 |
| 4 | **-0.009409** | -0.178553 | -1.412964 | -4.751875 | -11.100138 | -135.051028 |

Table 5: Average LnL differences to the best-known ML tree. Experiments conducted on DNA MSAs from TreeBASE. Tree inferences conducted under the GTR+G model.

| Bucket | true | RAxML-NG 100 | RAxML-NG | IQ-TREE2 | FastTree2 | pars | BIONJ |
|---|---|---|---|---|---|---|---|
| 0 | **0.994083** | 0.993097 | 0.993097 | 0.987179 | 0.962525 | 0.789941 | 0.666667 |
| 1 | 0.999120 | 0.997361 | **1.000000** | 0.996482 | 0.840809 | 0.438874 | 0.353562 |
| 2 | **1.000000** | **1.000000** | **1.000000** | 0.991704 | 0.786576 | 0.327300 | 0.303922 |
| 3 | 0.998371 | 0.997557 | **0.999186** | 0.988599 | 0.863192 | 0.585505 | 0.546417 |
| 4 | 0.986441 | **0.989831** | 0.972881 | 0.932203 | 0.884746 | 0.708475 | 0.718644 |

Table 6: Proportion of datasets passing the AU test. Experiments conducted on DNA MSAs from TreeBASE. Tree inferences conducted under the GTR+G model.

| Bucket | RAxML-NG 100 | RAxML-NG | IQ-TREE2 | FastTree2 | pars | BIONJ |
|---|---|---|---|---|---|---|
| 0 | **0.000000** | **0.000000** | **0.000000** | 0.062500 | 0.181818 | 0.193548 |
| 1 | **0.000000** | **0.000000** | 0.018182 | 0.156863 | 0.294118 | 0.342857 |
| 2 | **0.000000** | 0.023256 | 0.080000 | 0.237900 | 0.390244 | 0.400000 |
| 3 | **0.000000** | 0.200000 | 0.296296 | 0.423077 | 0.571429 | 0.541667 |
| 4 | **0.000000** | 0.655914 | 0.708333 | 0.732143 | 0.814286 | 0.791667 |

Table 7: Median RF distances to the best-known ML tree. Experiments conducted on DNA MSAs from TreeBASE. Tree inferences conducted under the GTR+G model.

| Bucket | RAxML-NG 100 | RAxML-NG | IQ-TREE2 | FastTree2 | pars | BIONJ |
|---|---|---|---|---|---|---|
| 0 | **0.000000** | 0.000023 | 0.000431 | 0.007200 | 0.025732 | 0.032415 |
| 1 | **0.000000** | 0.000054 | 0.000684 | 0.026824 | 0.055142 | 0.072162 |
| 2 | **0.000000** | 0.000122 | 0.001559 | 0.046747 | 0.081580 | 0.092795 |
| 3 | **0.000000** | 0.000161 | 0.002389 | 0.049677 | 0.078978 | 0.080135 |
| 4 | **0.000000** | 0.000470 | 0.001830 | 0.021092 | 0.037634 | 0.039427 |

Table 8: Median NTDs to the best-known ML tree. Experiments conducted on DNA MSAs from TreeBASE. Tree inferences conducted under the GTR+G model.

| Bucket | RAxML-NG 100 | RAxML-NG | IQ-TREE2 | FastTree2 | pars | BIONJ |
|---|---|---|---|---|---|---|
| 0 | **0.000000** | -0.000018 | -0.003082 | -0.152531 | -3.821044 | -4.768735 |
| 1 | **0.000000** | -0.000134 | -0.007879 | -4.721562 | -26.206793 | -40.635283 |
| 2 | **0.000000** | -0.000288 | -0.012139 | -8.539874 | -36.805708 | -50.358292 |
| 3 | **0.000000** | -0.000049 | -0.005759 | -3.273724 | -13.042831 | -13.636309 |
| 4 | **0.000000** | -0.000015 | -0.002608 | -0.036512 | -1.157584 | -0.680670 |

Table 9: Median LnL differences to the best-known ML tree. Experiments conducted on DNA MSAs from TreeBASE. Tree inferences conducted under the GTR+G model.

| Bucket | RAxML-NG | IQ-TREE2 | FastTree2 | pars | BIONJ |
|---|---|---|---|---|---|
| 0 | **0.076049** | 0.078937 | 0.084481 | 0.140366 | 0.169908 |
| 1 | **0.154833** | 0.160878 | 0.164798 | 0.238408 | 0.312078 |
| 2 | **0.273518** | 0.283403 | 0.286388 | 0.367614 | 0.435184 |
| 3 | **0.532143** | 0.543973 | 0.540601 | 0.607305 | 0.630892 |
| 4 | 0.827147 | 0.831712 | **0.818929** | 0.851819 | 0.858071 |

Table 10: Average RF distances to the true tree. Experiments conducted on simulated DNA MSAs using RAxMLGroveScripts with the BONK gap insertion. Tree inferences conducted under the GTR+G model.

| Bucket | RAxML-NG | IQ-TREE2 | FastTree2 | pars | BIONJ |
|---|---|---|---|---|---|
| 0 | 0.008554 | **0.008129** | 0.010657 | 0.021892 | 0.042776 |
| 1 | **0.014797** | 0.015359 | 0.019622 | 0.038402 | 0.089339 |
| 2 | **0.028181** | 0.029659 | 0.036622 | 0.057319 | 0.112913 |
| 3 | **0.040858** | 0.042119 | 0.047147 | 0.062200 | 0.090170 |
| 4 | **0.038494** | 0.040536 | 0.042592 | 0.050503 | 0.065594 |

Table 11: Average NTDs to the true tree. Experiments conducted on simulated DNA MSAs using RAxMLGroveScripts with the BONK gap insertion. Tree inferences conducted under the GTR+G model.

| Bucket | RAxML-NG | IQ-TREE2 | FastTree2 | pars | BIONJ |
|---|---|---|---|---|---|
| 0 | **0.666131** | 0.455331 | -1.155782 | -36.837734 | -981.747869 |
| 1 | **2.309241** | -0.214729 | -3.110655 | -78.977373 | -2512.571430 |
| 2 | **6.278071** | 4.339701 | -4.283485 | -72.938845 | -1980.849497 |
| 3 | **7.294462** | 3.541980 | -6.268921 | -35.779040 | -377.152160 |
| 4 | **3.676913** | 3.100384 | 1.961088 | -1.084208 | -36.829113 |

Table 12: Average LnL differences to the true tree. Experiments conducted on simulated DNA MSAs using RAxMLGroveScripts with the BONK gap insertion. Tree inferences conducted under the GTR+G model.

| Bucket | true | RAxML-NG | IQ-TREE2 | FastTree2 | pars | BIONJ |
|---|---|---|---|---|---|---|
| 0 | 0.867683 | **0.885151** | 0.864523 | 0.858576 | 0.504181 | 0.630552 |
| 1 | 0.905310 | **0.979205** | 0.969110 | 0.875025 | 0.318191 | 0.409247 |
| 2 | 0.788448 | **0.991953** | 0.975143 | 0.819564 | 0.286660 | 0.366237 |
| 3 | 0.721842 | **0.981278** | 0.961664 | 0.852006 | 0.484101 | 0.534324 |
| 4 | 0.760623 | **0.943343** | 0.934844 | 0.893768 | 0.703966 | 0.764873 |

Table 13: Proportion of datasets passing the AU test. Experiments conducted on simulated DNA MSAs using RAxMLGroveScripts with the BONK gap insertion. Tree inferences conducted under the GTR+G model.

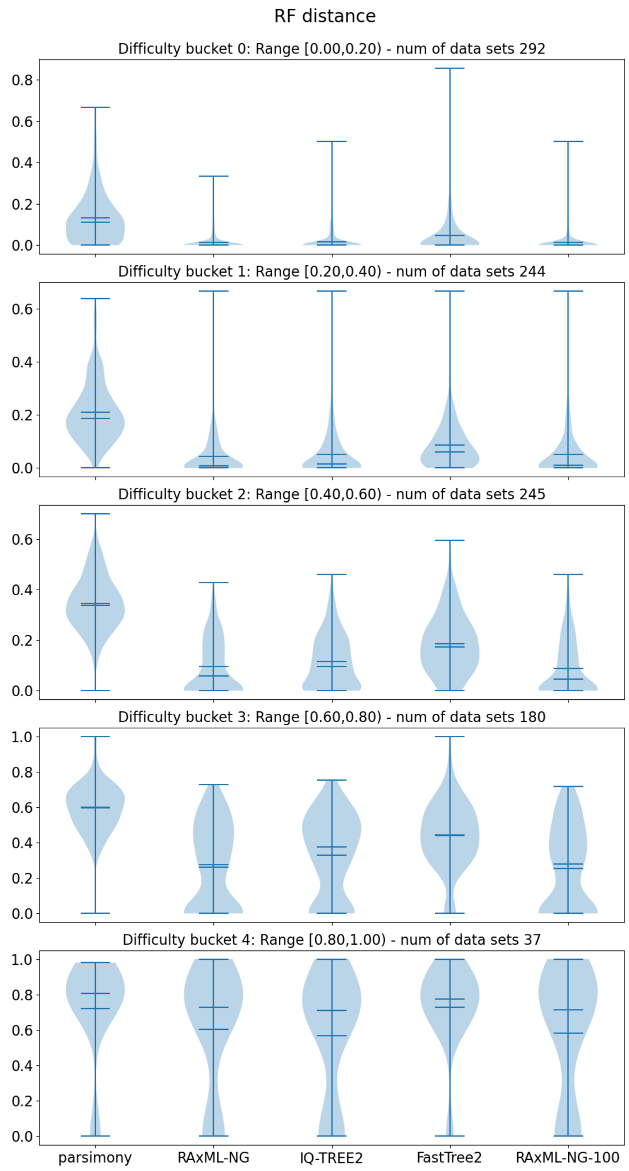


Figure 1: Average RF distances to the best-known-LnL tree. Experiments conducted on simulated DNA MSAs using RAxMLGroveScripts with the BONK gap insertion. Tree inferences conducted under the GTR+G model.

| Bucket | RAxML-NG 100 | RAxML-NG | IQ-TREE2 | FastTree2 | pars | BIONJ |
|---|---|---|---|---|---|---|
| 0 | **0.013904** | 0.014318 | 0.016747 | 0.047607 | 0.132616 | 0.151736 |
| 1 | 0.050548 | **0.043301** | 0.051065 | 0.087202 | 0.209920 | 0.254169 |
| 2 | **0.087737** | 0.095131 | 0.114953 | 0.185059 | 0.346945 | 0.364401 |
| 3 | **0.253674** | 0.262674 | 0.327127 | 0.437869 | 0.596901 | 0.569545 |
| 4 | 0.582498 | 0.603932 | **0.566675** | 0.726667 | 0.718843 | 0.802480 |

Table 14: Average RF distances to the best-known-LnL tree. Experiments conducted on simulated DNA MSAs using RAxMLGroveScripts with the BONK gap insertion. Tree inferences conducted under the GTR+G model.

| Bucket | RAxML-NG 100 | RAxML-NG | IQ-TREE2 | FastTree2 | pars | BIONJ |
|---|---|---|---|---|---|---|
| 0 | **0.000207** | 0.000583 | 0.000495 | 0.005160 | 0.019476 | 0.036255 |
| 1 | **0.000842** | 0.001118 | 0.004559 | 0.009747 | 0.030654 | 0.068618 |
| 2 | **0.000928** | 0.001940 | 0.007710 | 0.025523 | 0.059523 | 0.092628 |
| 3 | **0.001293** | 0.001579 | 0.021790 | 0.035560 | 0.064149 | 0.084920 |
| 4 | **0.015443** | 0.020773 | 0.020657 | 0.025271 | 0.024617 | 0.032529 |

Table 15: Average NTDs to the best-known-LnL tree. Experiments conducted on simulated DNA MSAs using RAxMLGroveScripts with the BONK gap insertion. Tree inferences conducted under the GTR+G model.

| Bucket | RAxML-NG 100 | RAxML-NG | IQ-TREE2 | FastTree2 | pars | BIONJ |
|---|---|---|---|---|---|---|
| 0 | **-0.001053** | -0.001670 | -0.003312 | -0.428189 | -37.840257 | -1931.385208 |
| 1 | **-1.053585** | -1.056790 | -6.290974 | -2.632134 | -67.556505 | -1542.676675 |
| 2 | **-0.001610** | -0.013393 | -1.073654 | -9.423050 | -85.763049 | -2210.946162 |
| 3 | **-0.002703** | -0.007521 | -3.543291 | -4.560207 | -26.372969 | -199.906827 |
| 4 | -0.000672 | **-0.000661** | -0.094462 | -0.641900 | -3.863487 | -3.459339 |

Table 16: Average LnL differences to the best-known-LnL tree. Experiments conducted on simulated DNA MSAs using RAxMLGroveScripts with the BONK gap insertion. Tree inferences conducted under the GTR+G model.

| Bucket | true | RAxML-NG 100 | RAxML-NG | IQ-TREE2 | FastTree2 | pars | BIONJ |
|---|---|---|---|---|---|---|---|
| 0 | 0.900685 | 0.890411 | **0.907534** | 0.890411 | **0.907534** | 0.510274 | 0.702055 |
| 1 | **0.987705** | 0.979508 | 0.983607 | 0.971311 | 0.913934 | 0.311475 | 0.434426 |
| 2 | 0.991837 | 0.991837 | **0.995918** | 0.987755 | 0.844898 | 0.285714 | 0.367347 |
| 3 | **0.977778** | 0.966667 | 0.966667 | 0.950000 | 0.888889 | 0.488889 | 0.527778 |
| 4 | **0.945946** | **0.945946** | 0.918919 | **0.945946** | **0.945946** | 0.648649 | 0.729730 |

Table 17: Proportion of datasets passing the AU test. Experiments conducted on simulated DNA MSAs using RAxMLGroveScripts with the BONK gap insertion. Tree inferences conducted under the GTR+G model. "True" tree selected based on the best-known-LnL.

### 3.2 Amino Acid MSAs

In this section we present the numerical results of experiments conducted on simulated and empirical amino acid (AA) MSAs using RAxMLGroveScripts (RGS) and TreeBASE databases respectively. For the simulations using RGS, we used the same data selection criteria as for the DNA data (see main text). Thus, we selected datasets with a number of taxa below 799 and a number of site patterns below 5433. We further filtered all datasets with the "AUTO" substitution model (which is an option in RAxML8). The users inferring trees on AA MSAs used a larger variety of substitution models, which we also used for our simulations. The resulting composition of substitution models for our simulations is as follows: 26.7% LG+G [15], 25.8% JTT+G [12], 25.6% Dayhoff+G [2], 7.2% WAG+G [31], 4.2% Blosum62+G [9], 2.2% VT+G [21], 1.7% GTR+G [27], 1.7% mtMAM+G [32], 4.9% others. In order to simplify our setup, and for the fact that FastTree2 does not support all of the mentioned substitution models, we inferred all the trees for our experiments on AA data under the LG+G model, since it is a commonly used model in the field and is supported by all of the analyzed tools. Numerical results of the experiments are shown in Tables 25 to 28.

For the experiments on TreeBASE AA data, we selected all MSAs from TreeBASE that we could automatically identify as AA MSAs. That is MSAs which contained characters besides A, C, G, T, N, "-" were automatically assumed to be AA MSAs. Numerical results of the experiments are shown in Tables 18 to 24.

## 4 AliSim Simulations with Superimposed Gaps

As mentioned in the main manuscript, we conducted an additional experiment on 5000 simulated MSAs using TreeBASE datasets (mentioned in the Section on the differences between simulated and empirical data). We used the AliSim mimick function, which first simulates an MSA, and then superimposes the gap pattern from a reference MSA to the simulated one. The results of these

| Bucket | RAxML-NG 100 | RAxML-NG | IQ-TREE2 | FastTree2 | pars | BIONJ |
|---|---|---|---|---|---|---|
| 0 | 0.005975 | **0.004944** | 0.019069 | 0.113320 | 0.264049 | 0.951243 |
| 1 | **0.010299** | 0.032119 | 0.122952 | 0.270034 | 0.471135 | 0.983838 |
| 2 | **0.020977** | 0.105037 | 0.286797 | 0.377194 | 0.571332 | 0.991708 |
| 3 | 0.139167 | **0.128810** | 0.356778 | 0.418147 | 0.584661 | 0.973627 |
| 4 | 0.391304 | **0.305970** | 0.771902 | 0.831603 | 0.860156 | 0.992538 |

Table 18: Average RF distances to the best-known ML tree. Experiments conducted on AA MSAs from TreeBASE. Tree inferences conducted under the LG+G model.

| Bucket | RAxML-NG 100 | RAxML-NG | IQ-TREE2 | FastTree2 | pars | BIONJ |
|---|---|---|---|---|---|---|
| 0 | **0.000888** | 0.001125 | 0.008759 | 0.029825 | 0.068827 | 0.477492 |
| 1 | **0.003439** | 0.010450 | 0.079744 | 0.102709 | 0.176203 | 0.494689 |
| 2 | **0.002575** | 0.028805 | 0.130146 | 0.132335 | 0.192789 | 0.504576 |
| 3 | **0.000366** | 0.006200 | 0.087818 | 0.095678 | 0.137019 | 0.621796 |
| 4 | 0.000371 | **0.000083** | 0.015524 | 0.032456 | 0.023454 | 0.469816 |

Table 19: Average NTDs to the best-known ML tree. Experiments conducted on AA MSAs from TreeBASE. Tree inferences conducted under the LG+G model.

| Bucket | RAxML-NG 100 | RAxML-NG | IQ-TREE2 | FastTree2 | pars | BIONJ |
|---|---|---|---|---|---|---|
| 0 | **-0.003656** | -0.008796 | -42.991280 | -15.361356 | -131.167824 | -28665.275962 |
| 1 | **-0.146885** | -0.422171 | -463.654283 | -207.520547 | -788.918041 | -27012.838108 |
| 2 | **-0.030886** | -12.653854 | -620.516140 | -483.645859 | -1194.960082 | -39399.725481 |
| 3 | **-0.000516** | -0.165212 | -140.614102 | -30.905465 | -79.708932 | -4196.876357 |
| 4 | **0.000000** | -0.000012 | -0.379365 | -4.345182 | -2.276281 | -206.054192 |

Table 20: Average LnL differences to the best-known ML tree. Experiments conducted on AA MSAs from TreeBASE. Tree inferences conducted under the LG+G model.

| Bucket | true | RAxML-NG 100 | RAxML-NG | IQ-TREE2 | FastTree2 | pars | BIONJ |
|---|---|---|---|---|---|---|---|
| 0 | **0.998016** | 0.992063 | 0.996032 | 0.984127 | 0.932540 | 0.484127 | 0.033730 |
| 1 | **1.000000** | **1.000000** | **1.000000** | 0.876543 | 0.845679 | 0.172840 | 0.024691 |
| 2 | **1.000000** | **1.000000** | **1.000000** | 0.640351 | 0.605263 | 0.096491 | 0.000000 |
| 3 | **1.000000** | **1.000000** | **1.000000** | 0.814815 | 0.925926 | 0.481481 | 0.000000 |
| 4 | **0.500000** | **0.500000** | **0.500000** | **0.500000** | **0.500000** | **0.500000** | 0.000000 |

Table 21: Proportion of datasets passing the AU test. Experiments conducted on AA MSAs from TreeBASE. Tree inferences conducted under the LG+G model.

| Bucket | RAxML-NG 100 | RAxML-NG | IQ-TREE2 | FastTree2 | pars | BIONJ |
|---|---|---|---|---|---|---|
| 0 | **0.000000** | **0.000000** | **0.000000** | 0.086032 | 0.258246 | 1.000000 |
| 1 | **0.000000** | **0.000000** | 0.005906 | 0.212406 | 0.436698 | 1.000000 |
| 2 | **0.000000** | 0.046886 | 0.279100 | 0.362231 | 0.568437 | 1.000000 |
| 3 | **0.000000** | 0.028571 | 0.400000 | 0.428571 | 0.592000 | 0.984615 |
| 4 | 0.391304 | **0.305970** | 0.771902 | 0.831603 | 0.860156 | 0.992538 |

Table 22: Median RF distances to the best-known ML tree. Experiments conducted on AA MSAs from TreeBASE. Tree inferences conducted under the LG+G model.

| Bucket | RAxML-NG 100 | RAxML-NG | IQ-TREE2 | FastTree2 | pars | BIONJ |
|---|---|---|---|---|---|---|
| 0 | **0.000110** | 0.000166 | 0.000200 | 0.013691 | 0.055178 | 0.492960 |
| 1 | **0.000015** | 0.000184 | 0.000745 | 0.057597 | 0.127928 | 0.492484 |
| 2 | **0.000000** | 0.001105 | 0.086791 | 0.091206 | 0.151287 | 0.511531 |
| 3 | **0.000068** | 0.000112 | 0.003017 | 0.069575 | 0.123382 | 0.642169 |
| 4 | 0.000371 | **0.000083** | 0.015524 | 0.032456 | 0.023454 | 0.469816 |

Table 23: Median NTDs to the best-known ML tree. Experiments conducted on AA MSAs from TreeBASE. Tree inferences conducted under the LG+G model.

| Bucket | RAxML-NG 100 | RAxML-NG | IQ-TREE2 | FastTree2 | pars | BIONJ |
|---|---|---|---|---|---|---|
| 0 | **-0.000087** | -0.000171 | -0.000169 | -0.819377 | -19.778971 | -4057.572241 |
| 1 | **-0.000003** | -0.000147 | -0.001765 | -14.637480 | -142.175765 | -9074.112590 |
| 2 | **0.000000** | -0.002542 | -7.181033 | -44.588745 | -307.215707 | -6462.620202 |
| 3 | **0.000000** | -0.000023 | -0.169024 | -6.821750 | -35.799429 | -1416.253060 |
| 4 | **0.000000** | -0.000012 | -0.379365 | -4.345182 | -2.276281 | -206.054192 |

Table 24: Median LnL differences to the best-known ML tree. Experiments conducted on AA MSAs from TreeBASE. Tree inferences conducted under the LG+G model.

| Bucket | RAxML-NG | IQ-TREE2 | FastTree2 | pars | BIONJ |
|---|---|---|---|---|---|
| 0 | 0.076098 | **0.075591** | 0.087817 | 0.199092 | 0.227806 |
| 1 | **0.114526** | 0.120681 | 0.134814 | 0.285100 | 0.279370 |
| 2 | **0.281606** | 0.288773 | 0.301711 | 0.466967 | 0.442058 |
| 3 | **0.556382** | 0.565192 | 0.573946 | 0.675032 | 0.668239 |
| 4 | 0.915146 | 0.914764 | **0.909523** | 0.927640 | 0.930747 |

Table 25: Average RF distances to the true tree. Experiments conducted on simulated AA MSAs using RAxMLGroveScripts with the BONK gap insertion. Tree inferences conducted under the LG+G model.

| Bucket | RAxML-NG | IQ-TREE2 | FastTree2 | pars | BIONJ |
|---|---|---|---|---|---|
| 0 | 0.020481 | **0.019810** | 0.023747 | 0.058532 | 0.079900 |
| 1 | 0.033699 | **0.033532** | 0.046589 | 0.096533 | 0.083415 |
| 2 | **0.092865** | 0.099444 | 0.109525 | 0.170660 | 0.151540 |
| 3 | **0.202063** | 0.207404 | 0.222943 | 0.261865 | 0.248270 |
| 4 | **0.024848** | 0.026133 | 0.027048 | 0.045843 | 0.031756 |

Table 26: Average NTDs to the true tree. Experiments conducted on simulated AA MSAs using RAxMLGroveScripts with the BONK gap insertion. Tree inferences conducted under the LG+G model.

| Bucket | RAxML-NG | IQ-TREE2 | FastTree2 | pars | BIONJ |
|---|---|---|---|---|---|
| 0 | 1.864011 | **69.881652** | 61.302897 | -81.760223 | -714.825861 |
| 1 | **4.847070** | 2.799627 | -23.879370 | -497.057881 | -1026.885604 |
| 2 | **21.681497** | 11.633129 | -13.064578 | -337.981340 | -372.172342 |
| 3 | **37.264278** | 31.602801 | 7.940814 | -164.556130 | -196.419605 |
| 4 | 6.144371 | **6.557440** | 5.589291 | 1.029950 | -3.787936 |

Table 27: Average LnL differences to the true tree. Experiments conducted on simulated AA MSAs using RAxMLGroveScripts with the BONK gap insertion. Tree inferences conducted under the LG+G model.

| Bucket | true | RAxML-NG | IQ-TREE2 | FastTree2 | pars | BIONJ |
|---|---|---|---|---|---|---|
| 0 | 0.939891 | 0.994536 | **0.997268** | 0.882514 | 0.224044 | 0.308743 |
| 1 | 0.763578 | **0.990415** | 0.913738 | 0.738019 | 0.047923 | 0.083067 |
| 2 | 0.518919 | **0.994595** | 0.848649 | 0.648649 | 0.064865 | 0.113514 |
| 3 | 0.373913 | **0.973913** | 0.895652 | 0.626087 | 0.104348 | 0.269565 |
| 4 | 0.380952 | **0.666667** | 0.619048 | **0.666667** | 0.380952 | 0.428571 |

Table 28: Proportion of datasets passing the AU test. Experiments conducted on simulated AA MSAs using RAxMLGroveScripts with the BONK gap insertion. Tree inferences conducted under the LG+G model.

experiments are shown in Figure 2.

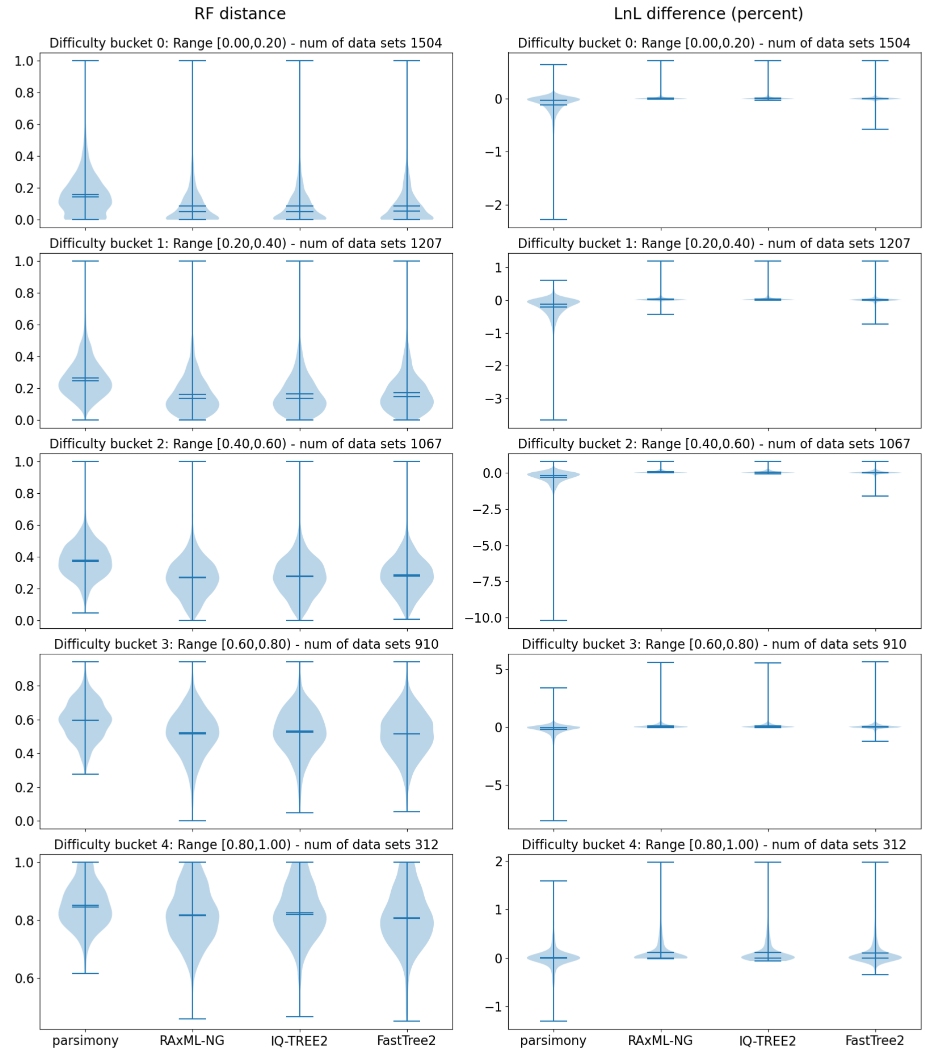


Figure 2: Relative log-likelihood (LnL) score differences, and RF-distances to the true tree of all inferred trees on simulated MSAs based on TreeBASE data (with superimposed gaps).

# 5 Used Tools

In addition to the tools mentioned in the main manuscript, our implementations of the experimental scripts re-use components from the following libraries and tools: BioPython [1, 26], draw.io (https://www.drawio.com/), Google Charts (http://developers.google.com/chart/), ETE 3 [10], Matplotlib [11], Numpy [7], Pandas [24], and SciPy [29].

# References


[1] Peter JA Cock, Tiago Antao, Jeffrey T Chang, Brad A Chapman, Cymon J Cox, Andrew Dalke, Iddo Friedberg, Thomas Hamelryck, Frank Kauff, Bartek Wilczynski, et al. Biopython: freely available python tools for computational molecular biology and bioinformatics. *Bioinformatics*, 25 (11):1422–1423, 2009.

[2] Margaret O Dayhoff. A model of evolutionary change in proteins. *Atlas of protein sequence and structure*, 5:89–99, 1972.

[3] Walter M Fitch. Toward defining the course of evolution: minimum change for a specific tree topology. *Systematic Biology*, 20(4):406–416, 1971.

[4] William Fletcher and Ziheng Yang. INDELible: A Flexible Simulator of Biological Sequence Evolution. *Molecular Biology and Evolution*, 26(8): 1879–1888, 05 2009. ISSN 0737-4038. doi: 10.1093/molbev/msp098. URL `https://doi.org/10.1093/molbev/msp098`.

[5] Olivier Gascuel. Bionj: an improved version of the nj algorithm based on a simple model of sequence data. *Molecular biology and evolution*, 14(7): 685–695, 1997.

[6] Julia Haag, Dimitri Höhler, Ben Bettisworth, and Alexandros Stamatakis. From easy to hopeless—predicting the difficulty of phylogenetic analyses. *Molecular biology and evolution*, 39(12):msac254, 2022.

[7] Charles R. Harris, K. Jarrod Millman, Stéfan J. van der Walt, Ralf Gommers, Pauli Virtanen, David Cournapeau, Eric Wieser, Julian Taylor, Sebastian Berg, Nathaniel J. Smith, Robert Kern, Matti Picus, Stephan Hoyer, Marten H. van Kerkwijk, Matthew Brett, Allan Haldane, Jaime Fernández del Río, Mark Wiebe, Pearu Peterson, Pierre Gérard-Marchant, Kevin Sheppard, Tyler Reddy, Warren Weckesser, Hameer Abbasi, Christoph Gohlke, and Travis E. Oliphant. Array programming with NumPy. *Nature*, 585(7825):357–362, September 2020. doi: 10.1038/s41586-020-2649-2. URL `https://doi.org/10.1038/s41586-020-2649-2`.

[8] Tim Head, Manoj Kumar, Holger Nahrstaedt, Gilles Louppe, and Iaroslav Shcherbatyi. scikit-optimize/scikit-optimize, October 2021. URL `https://doi.org/10.5281/zenodo.5565057`.

[9] Steven Henikoff and Jorja G Henikoff. Amino acid substitution matrices from protein blocks. *Proceedings of the National Academy of Sciences*, 89 (22):10915–10919, 1992.

[10] Jaime Huerta-Cepas, François Serra, and Peer Bork. Ete 3: reconstruction, analysis, and visualization of phylogenomic data. *Molecular biology and evolution*, 33(6):1635–1638, 2016.

[11] J. D. Hunter. Matplotlib: A 2d graphics environment. *Computing in Science & Engineering*, 9(3):90–95, 2007. doi: 10.1109/MCSE.2007.55.

[12] David T Jones, William R Taylor, and Janet M Thornton. The rapid generation of mutation data matrices from protein sequences. *Bioinformatics*, 8(3):275–282, 1992.

[13] Alexey M Kozlov, Diego Darriba, Tomáš Flouri, Benoit Morel, and Alexandros Stamatakis. RAxML-NG: a fast, scalable and user-friendly tool for maximum likelihood phylogenetic inference. *Bioinformatics*, 35(21):4453–4455, 05 2019. ISSN 1367-4803. doi: 10.1093/bioinformatics/btz305. URL `https://doi.org/10.1093/bioinformatics/btz305`.

[14] Harold J Kushner. A new method of locating the maximum point of an arbitrary multipeak curve in the presence of noise. 1964.

[15] Si Quang Le and Olivier Gascuel. An improved general amino acid replacement matrix. *Molecular biology and evolution*, 25(7):1307–1320, 2008.

[16] Eli Levy Karin, Dafna Shkedy, Haim Ashkenazy, Reed A Cartwright, and Tal Pupko. Inferring rates and length-distributions of indels using approximate bayesian computation. *Genome biology and evolution*, 9(5):1280–1294, 2017.

[17] Gil Loewenthal, Dana Rapoport, Oren Avram, Asher Moshe, Elya Wygoda, Alon Itzkovitch, Omer Israeli, Dana Azouri, Reed A Cartwright, Itay Mayrose, et al. A probabilistic model for indel evolution: differentiating insertions from deletions. *Molecular biology and evolution*, 38(12):5769–5781, 2021.

[18] Nhan Ly-Trong, Suha Naser-Khdour, Robert Lanfear, and Bui Quang Minh. Alisim: A fast and versatile phylogenetic sequence simulator for the genomic era. *Molecular Biology and Evolution*, 39(5):msac092, 2022.

[19] Bui Quang Minh, Heiko A Schmidt, Olga Chernomor, Dominik Schrempf, Michael D Woodhams, Arndt Von Haeseler, and Robert Lanfear. Iq-tree 2: new models and efficient methods for phylogenetic inference in the genomic era. *Molecular biology and evolution*, 37(5):1530–1534, 2020.

[20] Jonas Mockus, Vytautas Tiesis, and Antanas Zilinskas. The application of bayesian methods for seeking the extremum. *Towards global optimization*, 2(117-129):2, 1978.

[21] Tobias Müller and Martin Vingron. Modeling amino acid replacement. *Journal of Computational Biology*, 7(6):761–776, 2000.

[22] W. H. Piel, L. Chan, M. J. Dominus, J. Ruan, R. A. Vos, and V. Tannen. TreeBASE v. 2: A Database of Phylogenetic Knowledge. *e-BioSphere 2009*, 2009.

[23] Morgan N Price, Paramvir S Dehal, and Adam P Arkin. FastTree 2–approximately maximum-likelihood trees for large alignments. *PloS one*, 5 (3):e9490, 2010.

[24] Jeff Reback, Wes McKinney, jbrockmendel, Joris Van den Bossche, Tom Augspurger, Phillip Cloud, gfyoung, Sinhrks, Simon Hawkins, Matthew Roeschke, Adam Klein, Terji Petersen, Jeff Tratner, Chang She, William Ayd, Shahar Naveh, Marc Garcia, Jeremy Schendel, Andy Hayden, Daniel Saxton, Vytautas Jancauskas, Ali McMaster, Pietro Battiston, Skipper Seabold, chris b1, h vetinari, Kaiqi Dong, Stephan Hoyer, Wouter Overmeire, and Marco Gorelli. pandas-dev/pandas: Pandas 1.1.4, October 2020. URL `https://doi.org/10.5281/zenodo.4161697`.

[25] Donald B Rubin. Bayesianly justifiable and relevant frequency calculations for the applied statistician. *The Annals of Statistics*, pages 1151–1172, 1984.

[26] Eric Talevich, Brandon M Invergo, Peter JA Cock, and Brad A Chapman. Bio. phylo: a unified toolkit for processing, analyzing and visualizing phylogenetic trees in biopython. *BMC bioinformatics*, 13(1):1–9, 2012.

[27] Simon Tavaré. Some probabilistic and statistical problems in the analysis of dna sequences. *Lect Math Life Sci (Am Math Soc)*, 17:57–86, 1986.

[28] Simon Tavaré, David J Balding, Robert C Griffiths, and Peter Donnelly. Inferring coalescence times from dna sequence data. *Genetics*, 145(2):505–518, 1997.

[29] Pauli Virtanen, Ralf Gommers, Travis E. Oliphant, Matt Haberland, Tyler Reddy, David Cournapeau, Evgeni Burovski, Pearu Peterson, Warren Weckesser, Jonathan Bright, Stéfan J. van der Walt, Matthew Brett, Joshua Wilson, K. Jarrod Millman, Nikolay Mayorov, Andrew R. J. Nelson, Eric Jones, Robert Kern, Eric Larson, C J Carey, İlhan Polat, Yu Feng, Eric W. Moore, Jake VanderPlas, Denis Laxalde, Josef Perktold, Robert Cimrman, Ian Henriksen, E. A. Quintero, Charles R. Harris, Anne M. Archibald, Antônio H. Ribeiro, Fabian Pedregosa, Paul van Mulbregt, and SciPy 1.0 Contributors. SciPy 1.0: Fundamental Algorithms for Scientific Computing in Python. *Nature Methods*, 17:261–272, 2020. doi: 10.1038/s41592-019-0686-2.

[30] Rutger A Vos, James P Balhoff, Jason A Caravas, Mark T Holder, Hilmar Lapp, Wayne P Maddison, Peter E Midford, Anurag Priyam, Jeet Sukumaran, Xuhua Xia, et al. Nexml: rich, extensible, and verifiable representation of comparative data and metadata. *Systematic biology*, 61(4):675–689, 2012.

[31] Simon Whelan and Nick Goldman. A general empirical model of protein evolution derived from multiple protein families using a maximum-likelihood approach. *Molecular biology and evolution*, 18(5):691–699, 2001.

[32] Ziheng Yang, Rasmus Nielsen, and Masami Hasegawa. Models of amino acid substitution and applications to mitochondrial protein evolution. *Molecular biology and evolution*, 15(12):1600–1611, 1998.

[33] AG Zhilinskas. Single-step bayesian search method for an extremum of functions of a single variable. *Cybernetics*, 11(1):160–166, 1975.
